# Mild Therapeutic Hypothermia Alleviated Myocardial Ischemia/Reperfusion Injury via Targeting SLC25A10 to Suppress Mitochondrial Apoptosis

**DOI:** 10.1007/s12265-024-10503-z

**Published:** 2024-04-03

**Authors:** Senlin Ma, Yun Song, Yanxin Xu, Chao Wang, Yifan Yang, Yanchao Zheng, Qiuxin Lu, Qingjiang Chen, Jian Wu, Bin Wang, Mingquan Chen

**Affiliations:** 1 Department of Emergency, Huashan Hospital, Fudan University, Shanghai, 200040 China; 2 Department of Pharmacy, Huashan Hospital, Fudan University, Shanghai, 200040 China

**Keywords:** Mild therapeutic hypothermia, Myocardial ischemia/reperfusion injury, SLC25A10, Mitochondrial apoptosis pathway

## Abstract

Myocardial ischemia/reperfusion injury (MI/RI) is identified as a severe vascular emergency, and the treatment strategy of MI/RI still needs further improvement. The present study aimed to investigate the potential effects of mild therapeutic hypothermia (MTH) on MI/RI and underlying mechanisms. In ischemia/reperfusion (I/R) rats, MTH treatment significantly improved myocardial injury, attenuated myocardial infarction, and inhibited the mitochondrial apoptosis pathway. The results of proteomics identified SLC25A10 as the main target of MTH treatment. Consistently, SLC25A10 expressions in I/R rat myocardium and hypoxia and reoxygenation (H/R) cardiomyocytes were significantly suppressed, which was effectively reversed by MTH treatment. In H/R cardiomyocytes, MTH treatment significantly improved cell injury, mitochondrial dysfunction, and inhibited the mitochondrial apoptosis pathway, which were partially reversed by SLC25A10 deletion. These findings suggested that MTH treatment could protect against MI/RI by modulating SLC25A10 expression to suppress mitochondrial apoptosis pathway, providing new theoretical basis for clinical application of MTH treatment for MI/RI.

**Graphical Abstract:**

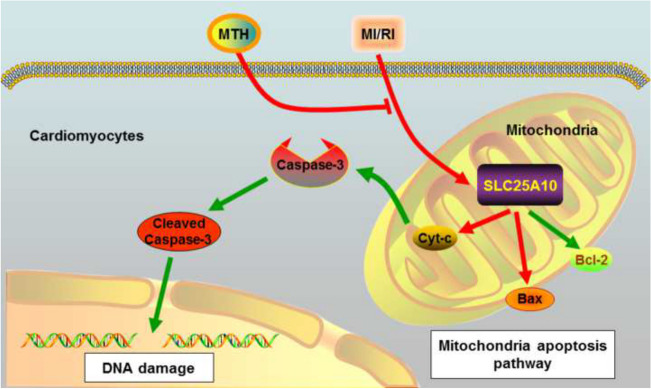

**Supplementary Information:**

The online version contains supplementary material available at 10.1007/s12265-024-10503-z.

## Introduction

Ischemic heart disease (IHD) is one of the leading causes of death worldwide, accounting for about 9 million deaths annually [1]. Acute myocardial infarction (AMI), the most life-threatening complication of IHD, severely blocked blood flow to the myocardium due to acute thrombotic occlusion of the coronary artery, finally resulting in the lack of oxygen and nutrients and cardiomyocyte death [2]. Currently, for AMI treatment, primary percutaneous coronary intervention or thrombolytic therapy is the main therapeutic intervention, which could effectively reduce myocardial infarct size and mortality [3]. However, myocardial reperfusion may cause injury to the myocardium, known as myocardial ischemia reperfusion injury (MI/RI) that contributes to the irreversible damage of ischemic tissues, as well as final heart failure [3]. Despite the continuous progress in understanding mechanisms of MI/RI, the treatment of MI/RI remains disappointing in clinical practice.

Mitochondrial dysfunction induced by MI/RI is known as the critical factor of cell death because mitochondria generate 6 kg/day of ATP to allow the heart to beat 100,000 times a day [4]. In particular, mitochondrial apoptosis pathway was reported to participate in multiple cardiovascular diseases, including MI/RI and heart failure [5]. Generally, mitochondrial apoptosis pathway was initiated by intracellular stimuli, such as oxidative stress, hypoxia, and nutrient deprivation, thus leading to abnormal activations of Bcl-2 family and release of cytochrome C (Cyt-C) from mitochondria, which triggers the caspase cascade and final cardiomyocyte apoptosis [6, 7]. Therefore, preventing mitochondrial dysfunction may be a potential therapeutic strategy for cardioprotection.

Mild therapeutic hypothermia (MTH, 32-34 °C) has been an established therapeutic method for the improvement of neurologic outcome after cardiac arrest and successful resuscitation for decades [8]. MTH treatment could diminish the demand of brain tissue for oxygen and energy by temporarily reducing the metabolic rate, thus preventing mitochondrial damage and oxidative stress in neurons, and ultimately reducing neuronal apoptosis [9]. For example, the lytic cocktail composed of promethazine, chlorpromazine, and pethidine hydrochloride can reduce the body’s response to various pathological stimuli and improve the tolerance of various tissues to hypoxia [10]. Therefore, MTH treatment has been applied to decrease myocardial infarction size, and improve cardiac function in various animal models [11, 12]. Although it was reported that MTH treatment inhibited heart failure development, these findings are still quite limited and MTH treatment failed to reduce MI size in clinical trials due to uncertain mechanism [13, 14].

Therefore, more investigations are needed to confirm the beneficial effects of MTH on MI/RI and the underlying mechanisms. Using in vitro and in vivo models, we comprehensively explored the protective effect of MTH on MI/RI and the molecular mechanisms. Our findings provided new insights into the role of MTH in cardioprotection and suggested SLC25A10 as the therapeutic target of MTH treatment for MI/RI.

## Material and Methods

### Chemicals and Reagents

The promethazine (Cat#46682) and chlorpromazine (Cat#31679) were purchased from Sigma-Aldrich (St Louis, MO, USA). The pethidine hydrochloride was purchased from QingHai Pharm (Lot. No 211103-1, China). The commercial kits for creatine kinase (CK)-MB, cardiac troponin 1 (cTnl), and lactic dehydrogenase (LDH) were purchased from Nanjing Jiancheng Bioengineering Institute (Nanjing, China). The commercial kits for ROS, MDA, ATP, SOD, cell, and tissue mitochondria isolation were purchased from Beyotime Biotech (Shanghai, China). The hematoxylin-eosin (HE) solution and triphenyl tetrazolium chloride (TTC) staining were purchased from KangLang Biotech (Shanghai, China). DMEM/F12 culture medium and fetal bovine serum (FBS) were from Gibco (Grand Island, NY, USA). A human cardiomyocyte cell line (AC16) was obtained from the American Type Culture Collection (ATCC, USA). JC-1 (5,5′,6,6′-tetrachloro-1,1′,3,3′-tetraethylbenzimid azolcarbocyanine iodide) kit (Cat#CS-7539) and H2DCFDA (2′,7′-dichlorodihydrofluorescein diacetate) probe (Cat#CS-1148) were purchased from MedChemExpress (MCE, New Jersey, US). The antibodies of anti-Bcl-2 (Cat#15071), anti-Bax (Cat#5023), anti-cleaved-caspase-3 (Cat#9661), anti-Cyt-C (Cat#4280), anti-β-actin (Cat#3700), and HRP-labeled goat anti-Rabbit/Mouse IgG (H + L) were purchased from Cell Signaling Technology (CA, USA). The anti-SLC25A10 antibody (Cat#12086) and were purchased from Proteintech Group, Inc (Wuhan, Hubei, China).

### MI/RI Rat Model

Adult Sprague-Dawley (SD) rats (male, 220-250 g) were provided by the Experimental Animal Center of Fudan University. SD rats were housed at 25 °C under 60% relative humidity with a 12-h light/12-h dark cycle. SD rats were acclimatized for one week before the experiment with ample drinking water and food. All procedures were approved by the ethics committee of Department of Experimental Animal Science, Fudan University (2022JS Huashan Hospital-070).

The MI/RI rat model was established by ligating the left anterior descending (LAD) coronary artery for 30 min followed by 24 h of reperfusion. Briefly, SD rats were anesthetized by sodium pentobarbital (50 mg/kg) intraperitoneally. After the completion of tracheal intubation, 6-0 silk was used to ligate the LAD for 30 min, and then, reperfusion was allowed for another 24 h by relieving the LAD ligation. SD rats in sham group were treated with the same surgical procedures without the ligation of LAD.

### Experimental Groups

According to previous reports [15], SD rats were randomly divided into three groups (*n* = 12 per group, six rats for TTC staining and six rats for other examinations): (1) Sham; (2) I/R; (3) I/R + MTH. Sham, rats receiving vehicle (0.9% NaCl) without being subjected to I/R; I/R, rats receiving vehicle (0.9% NaCl) with being subjected to I/R; I/R + MH, rats receiving the injection of a dose of 3 ml/kg mild hypothermia mixture (50 mg promethazine + 50 mg chlorpromazine + 100 mg pethidine hydrochloride dissolved in 100 ml normal saline, IP) after the onset of ischemia. A second injection with one third of the original dose was added at 1 h after reperfusion to enhance the drugs’ effects. SD rats in the Sham group and I/R group were given equal volumes of vehicle at the same time. Rectal temperature of rats in each group was measured once an hour to confirm the cooling effects of hypothermia mixture. After receiving first injection for 2 h, the average temperature of rats in the MTH group kept at 36.5 °C, indicating the successful cooling effect of MTH treatment.

### Assessment of Cardiac Function and Myocardial Injury

Ventricular parameters of rats including left ventricular internal dimension at end-diastole (LVIDd) and left ventricular internal dimension at systole (LVIDs) were assessed using the Vevo2100 High-Resolution Imaging System (VisualSonics, Canada).

Serum samples were collected for the assessment of myocardial injury markers. CK-MB, LDH, and cTnl in serum were determined by commercial assay kits according to instructions.

### Histopathological Analysis

For HE staining, rat hearts were fixed in 10% formalin solution. After embedding in paraffin wax, the tissues were sliced to a 4-µm thickness, and then were stained with HE solution.

For TTC staining, rat hearts were cut vertically into 2 mm slices along the heart’s longitudinal axis, and placed in 1% TTC solution, then fixed in 4% paraformaldehyde solution. The TTC staining pictures were analyzed by Image J. The crimson area was identified as non-infarcted and grayish-white area as infarcted.

For immunohistochemistry, 4-μm thickness slide was deparaffinized in xylene, and then rehydrated in ethanol and deionized water. After heat-mediated antigen retrieval, slides were incubated with anti-SLC25A10 antibody at 4 °C overnight and then incubated with HRP-conjugated secondary antibody. Stained by hematoxylin, the slides were then stained with 3,3′-diaminobenzidine and photographed under a light microscope (Leica Microsystems, USA).

### Cell Treatments

To mimic myocardial I/R injury in vitro, AC16 cells were grown in glucose-free medium in an anaerobic chamber (95% N_2_ and 5% CO_2_) at 37 °C for 8 h. Then, AC16 cells were returned to normal glucose-containing medium (4.5 mg/mL) and grown in a normal incubator with 95% air and 5% CO_2_ for another 12 h. Based on previous reports [16], to establish MTH (33.5 °C) treatment for H/R cell, incubation temperature was decreased to 33.5 °C after the onset of hypoxia and maintained during simulated reperfusion. Control group and hypoxia/reperfusion (H/R) groups were maintained at 37 °C throughout the duration of the experiment.

### Nano-UHPLC-MS/MS Analysis

Total proteins of harvested hearts were extracted by lysis buffer. In brief, after calculation of concentration and purity of the samples, the samples were then digested with trypsin and the peptides were re-dissolved in solvent A (A: 0.1% formic acid in water), which were analyzed by Orbitrap Fusion LUMOS mass spectrometer (Thermo Fisher Scientific) coupled to an EASY-nanoLC 1200 system (Thermo Fisher Scientific, MA, USA)[17]. Raw data of DIA were processed and analyzed by Spectronaut 14 (Biognosys AG, Switzerland) with dDIA default settings. Retention time prediction type was set to dynamic iRT. The average top 3 filtered peptides which passed the 1% *Q* value cutoff were used to calculate the major group quantities. After one-way ANOVA test, different expressed proteins were selected if *p* < 0.05 and absolute fold change > 1.5. Kyoto encyclopedia of genes and genomes (KEGG) pathway analysis was performed via the “clusterProfiler” package in R/Bioconductor software to acquire the enriched biological process and KEGG pathway for subsequent analysis.

### Cell Transfections

AC16 cells were transfected with siRNA targeting SLC25A10 (GenePharma, Shanghai, China) to downregulate SLC25A10 expression according to the manufacturer′s instructions. Lipofectamine 3000 reagent (Sigma-Aldrich, St. Louis, MO, USA) was used to transfect NC plasmid and SLC25A10 overexpression plasmid into AC16 cells. At 48 h post-transfection, the cells were subjected to H/R and then used for further examinations.

### MTT Assay

The AC16 cells of each group were seeded in a 96-well culture plate at a density of 10^4^/well. After grown for 48 h and treatment, the supernatant of cells was replaced by MTT solution. The OD value at 490 nm was measured by multiscan spectrum. The cell viability rate was calculated as (OD value of experimental group/OD value of control group) × 100%.

### Detection of Cyt-C Secretion

To investigate the secretion of Cyt-C from mitochondria into the cytoplasm, mitochondria was removed by using the Cell Mitochondria Isolation Kit or the Tissue Mitochondria Isolation Kit (Beyotime, Shanghai, China) to obtain cytoplasm proteins in cardiomyocytes or myocardium according to the manufacturer′s instructions. Then, Cyt-C in cytoplasm was measured by western blot analysis as described below.

### Western Blot

The collected myocardial tissues or cardiomyocytes were placed into RIPA lysate containing a protease inhibitor, homogenized, and centrifuged. After collecting the supernatant, the protein was quantified by BCA kit and was used for SDS-PAGE. After the protein was transferred to the PVDF membrane, the membrane was blocked by 5% milk followed by incubation with primary antibody against SLC25A10 at 4 °C overnight. Next, after the membrane was incubated with corresponding HRP-linked secondary antibodies, the blots were developed using an enhanced chemiluminescence system, and Image J was used to analyze the density of blots.

### Measurement of Cellular ATP and Oxidative Stress

The cellular contents of ATP, ROS, MDA, and SOD activity were measured using commercial kits. All procedures were performed according to the manufacturer’s instructions. To visualize the generation of ROS, AC16 cells were stained with H2DCF-DA probe.

### JC-1 Staining and MitoSOX Staining

For JC-1 staining, mitochondrial membrane potential (MMP, Δψm) was determined using the JC-1 kit. AC16 cells were seeded on sterile cover glasses in 6-well plates and then incubated with JC-1 staining solution (2 μM) in PBS for 15 min at 37 °C. After staining, cells were visualized and photographed by a fluorescence microscopy.

For MitoSOX staining, AC16 cells were incubated with MitoSOX™ Red (3 μM) at 37 °C for 30 min, followed by washing twice. Fluorescence intensity was measured using Image J.

### Immunofluorescence Staining

AC16 cells were seeded on glass slides in 12-well plates and incubated as indicated for 48 h. After treatment, the slides were fixed in 4% paraformaldehyde for 30 min at 37 °C, and then permeabilized with 0.3% Triton X-100 for 15 min. Cells were then blocked with 3% BSA for 1 h at 37 °C followed by incubation with a primary antibody SLC25A10 at 4 °C overnight. Slices were incubated with a FITC-conjugated IgG for 1 h at 37 °C, and then labeled with DAPI for 5 min. Finally, slices were photographed under a fluorescence microscopy.

### Statistical Analysis

All the experimental data are expressed as the means ± SD and analyzed by Prism GraphPad 6.0 (San Diego, USA). The *t*-test was performed to measure the differences between the two groups and one-way analysis of variance (ANOVA) followed by a Dunnett’s test was performed to compare the differences among three or more groups. *p*-values < 0.05 were considered statistical significance.

## Results

### MTH Treatment Alleviated Myocardial Injury in I/R Rats

**Fig. 1:**
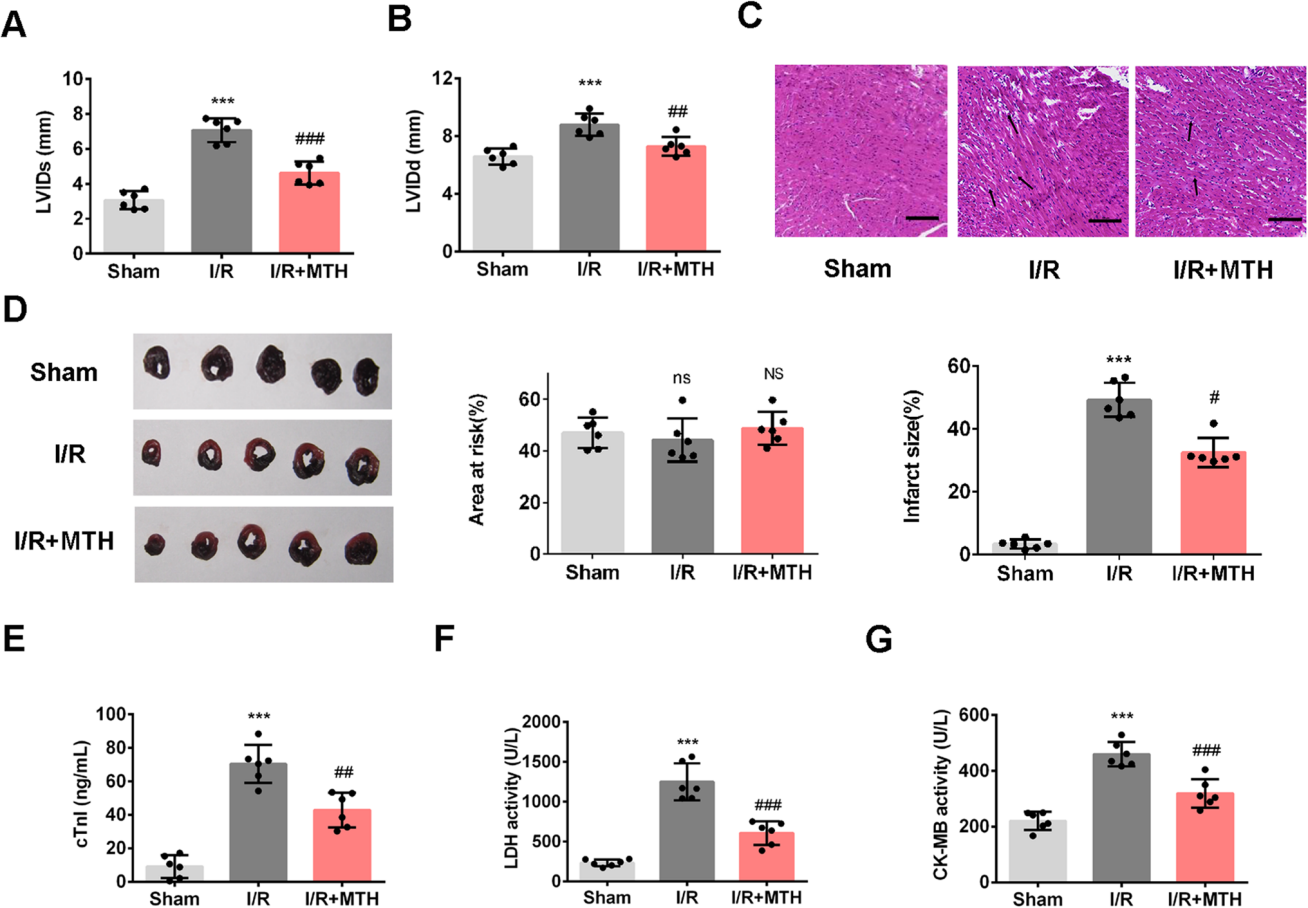
Effects of MTH treatment on myocardial injury in I/R rats. **A** LVIDs and **B** LVIDd were measured by transthoracic echocardiography. **C** Myocardial infarct size in rats was investigated by TTC staining and the representative photographs of TTC staining of each group. **D** Histological changes in the myocardial tissue were examined using H&E staining, scale bar: 100 μm. cTn1 content **E** in rat serum was examined by ELISA test. LDH activity **F** and CK-MB activity **G** in rat serum were examined by the commercial kits. **H** The expressions of mitochondrial apoptosis-related proteins, including Bcl-2, Bax, cleaved-caspase-3, and Cyt-C (in cytoplasm) were determined by western blot. Data shown are means ± SD, *n* = 6. ^**^P < 0.01 and ^***^P < 0.001 compared with Sham group; ^#^*P* < 0.05, ^##^*P* < 0.01, and ^###^*P* < 0.001 compared with I/R

To determine the effects of MTH on MI/RI, the cardiac function was evaluated by transthoracic echocardiography in rat models. As depicted in Fig. 1A–B, I/R rats exhibited significantly elevated LVIDs and LVIDd (*p* < 0.001), while MTH treatment significantly recovered LVIDs and LVIDd (*p* < 0.01) when compared to I/R rats, confirming the protective effects of MTH treatment on cardiac function in MI/RI. The results of TTC staining indicated an obvious increased MI area in I/R rats compared to the sham group (*p* < 0.001), while MTH treatment effectively reduced the MI area (*p* < 0.05; Fig. 1C). H&E staining further confirmed the damaged cardiac structures in I/R group, whereas the cardiac damage was partially relieved by MTH treatment compared to the I/R group (Fig. 1D). These results suggested that MTH treatment could protect against MI/RI. The serum markers for cardiac injury were examined by the commercial kits, including serum LDH activity (Fig. 1E), cTn1 content (Fig. 1F), and CK-MB activity (Fig. 1G). The results revealed significantly elevated serum levels of cardiac damage markers in I/R rats compared with sham group (*p* < 0.001), while MTH treatment significantly decreased the levels of these markers (*p* < 0.01).

Next, we tested the expressions of proteins in the mitochondria-mediated apoptotic pathway (Fig. 1H). The present data revealed that the cardiac expression of cleaved-caspase-3, Cyt-C (in cytoplasm), and Bax proteins were prominently increased in I/R rats and Bcl-2 was decreased relative to the sham group (*p* < 0.01). Notably, MTH treatment has reversed the abnormal expressions of these mitochondrial apoptosis-related proteins (*p* < 0.05). These data demonstrated that MTH treatment suppressed mitochondrial apoptosis pathway activated by MI/RI.

### SLC25A10 Might Be the Target of MTH Treatment as Revealed by Proteomics

**Fig. 2:**
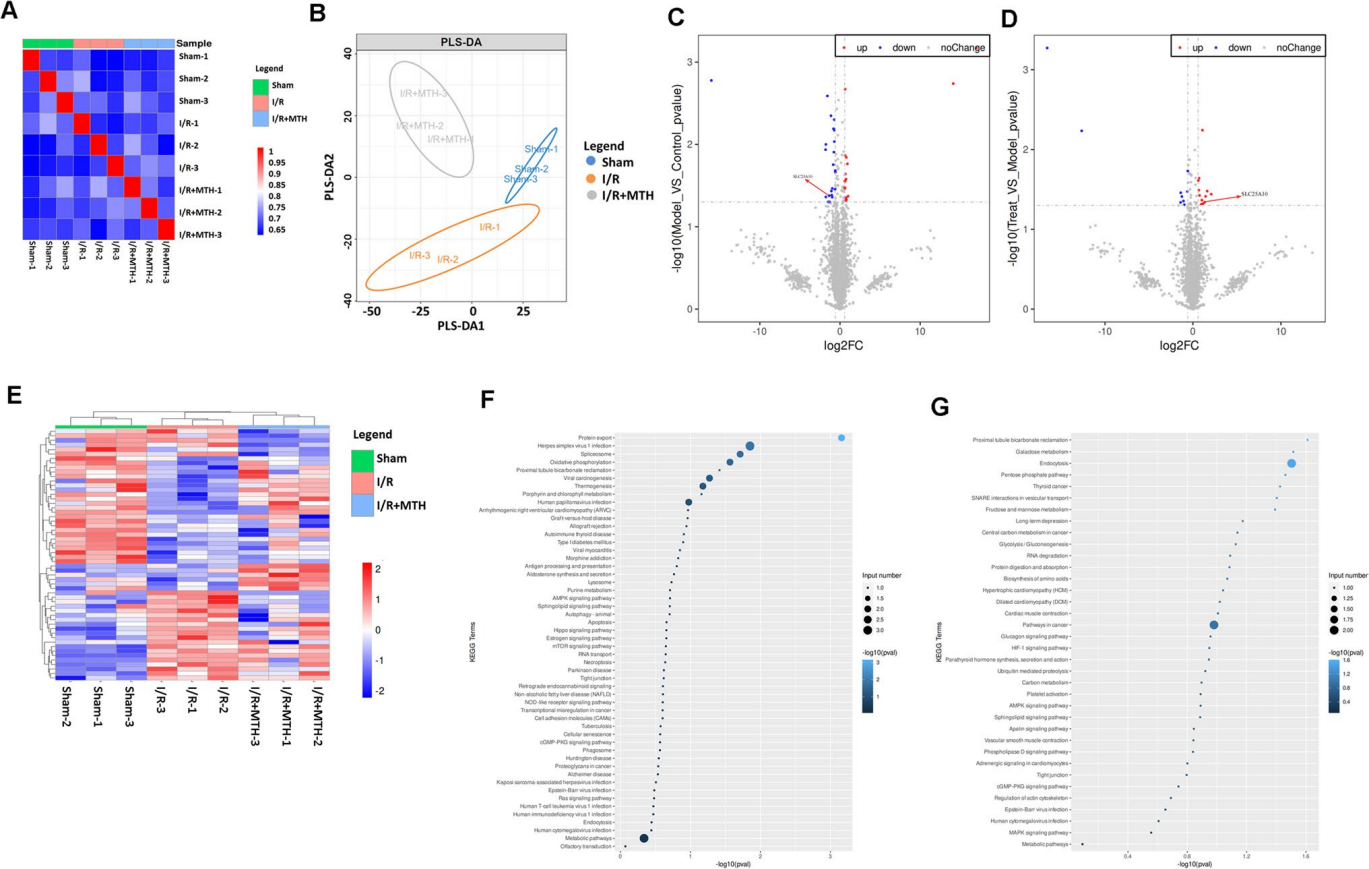
Potential targets of MTH treatment in MI/RI as revealed by proteomics. **A** The sample correlation between the sham, I/R, and I/R + MTH groups was calculated by the R language stats package cor function, and the sample correlation was visualized by the pheatmap package. **B** PLS-DA was calculated by MixOmics package for discriminant analysis. **C**, **D** Visualization analysis of differentially expressed proteins in sham vs I/R and I/R + MTH vs I/R using volcano map. **E** Visualization of the significant differentially expressed proteins among all comparison groups using unsupervised clustering heatmaps. **F**, **G** KEGG enrichment analysis of differentially expressed proteins in sham vs I/R and I/R + MTH vs I/R, respectively

To further elucidate the underlying mechanisms of MTH treatment in MI/RI, nano-UHPLC-MS/MS was performed by using myocardial tissues from sham, I/R and I/R + MTH groups. Before the expression difference analysis of protein samples, sample correlation and partial least squares discriminant analysis (PLS-DA) was analyzed to determine the repeatability and convergence of the samples, as to exclude severely outlier samples and ensure the stability and repeatability (Fig. 2A and B). With a criterion of *p*-value < 0.05 and fold changes ≥ 2, as shown in volcano plots and heat map, 60 significantly changed proteins were displayed in Fig. 2C–E. Moreover, the expression of 21 proteins was significantly changed in comparison between sham group and I/R group, while 39 proteins were significantly changed in comparison between I/R group and MTH treatment group (Fig. 2E; Supplementary table S1  and S2). In these differentially expressed proteins, SLC25A10 was dramatically inhibited in I/R group (*p* < 0.05), which was significantly rescued by MTH treatment (*p* < 0.05). Based on the results of KEGG analyses, nine signaling pathways were screened in the comparison of the sham group, I/R group, and I/R + MTH group, such as metabolic pathways, AMPK signaling pathway, Sphingolipid signaling pathway, and cGMP-PKG signaling pathway as showed in Fig. 2F and G. Therefore, based on the results of proteomics and the role of SLC25A10 in mitochondrial function and energy metabolism [18], we speculated that MTH treatment protected the heart from I/R injury possibly by regulating SLC25A10.

### MTH Treatment Upregulated SLC25A10 Expression In Vivo and In Vitro

**Fig. 3:**
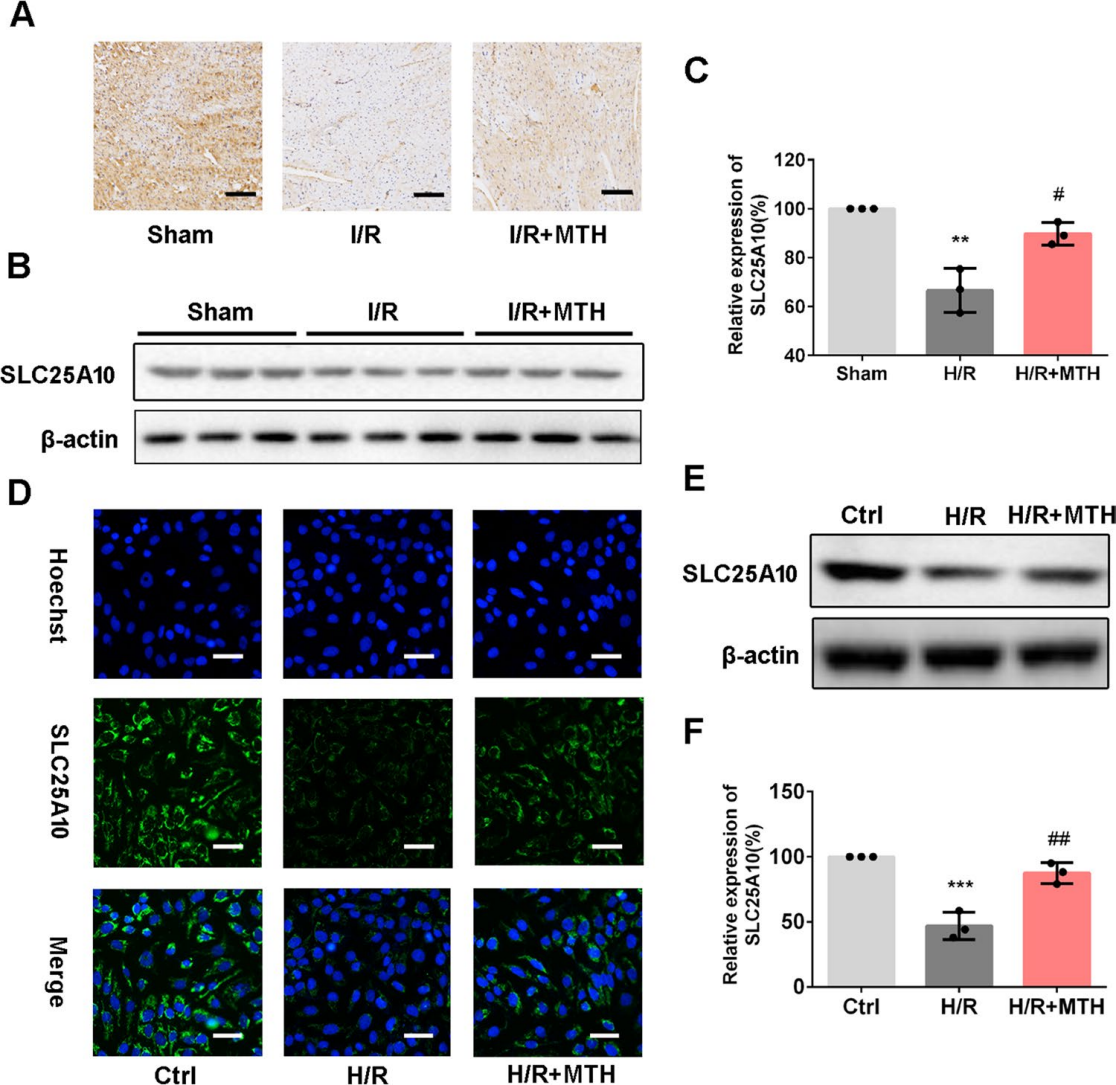
MTH treatment upregulated SLC25A10 in vivo and in vitro. **A** Immunohistochemistry of SLC25A10 in rat hearts, scale bar: 100 μm; **B**, **C** Western blot analysis of SLC25A10 in rat hearts. **D** SLC25A10 expression in AC16 cells was determined by immunofluorescence staining, scale bar: 50 μm. **E**, **F** Western blot analysis of SLC25A10 in AC16 cells. Data shown are means ± SD, *n* = 3. ^**^P < 0.01 and ^***^P < 0.001 compared with the Sham group; ^#^*P* < 0.05 and ^##^*P* < 0.01 compared with the I/R group

To determine whether SLC25A10 is functionally linked to the therapeutic effect of MTH treatment, we initially compared the expressions of SLC25A10 in myocardial tissues by immunohistochemistry (Fig. 3A) and western blot (Fig. 3B and C). It revealed that SLC25A10 expression was evidently decreased in myocardial tissues of I/R rats (*p* < 0.01), which was markedly reversed by MTH treatment (*p* < 0.05). Consistent with the animal data, hypoxia and reoxygenation (H/R) significantly inhibited SLC25A10 expression, which was restored by MTH treatment, as revealed by immunofluorescence (Fig. 3D) and western blot (Fig. 3E and F). These results indicated that SLC25A10 might be involved in the protective effect of MTH treatment on MI/RI.

### MTH-Induced Cardioprotection Was Partly Mediated by Upregulation of SLC25A10

**Fig. 4:**
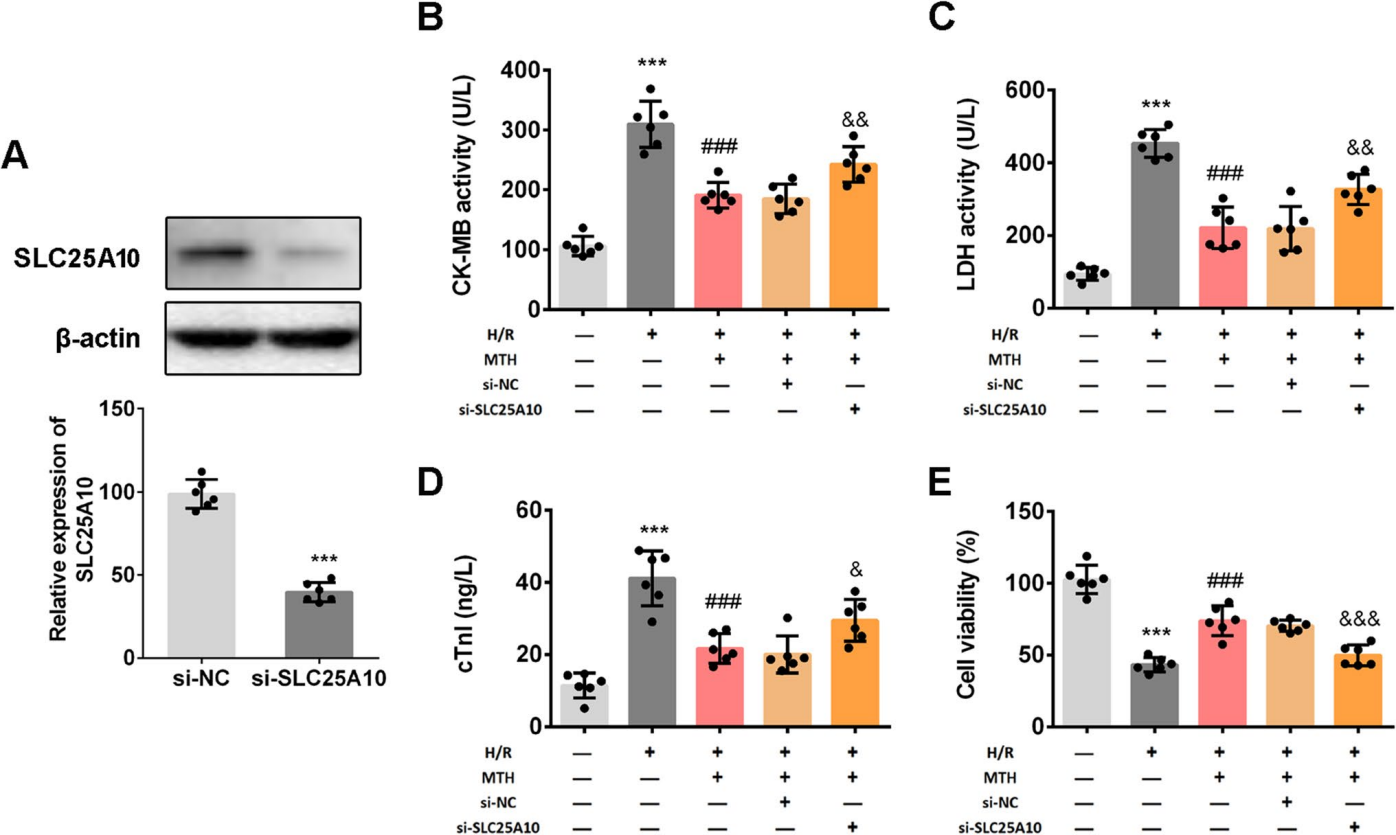
SLC25A10 was involved in MTH-induced protection in H/R cardiomyocyte. **A** SLC25A10 expression in AC16 cells was knockdown by siRNA transfection and was determined by western blot. CK-MB activity **B** and LDH activity **C** in serum were examined by commercial assay kits. Level of cTn1 in serum **D** was examined by ELISA test. **E** Cell viability of AC16 cells was measured by MTT assay. Data shown are means ± SD, *n* = 6. ^***^P < 0.001 compared with Ctrl group; ^###^*P* < 0.001 compared with the H/R group; ^&^*P* < 0.05, ^&&^*P* < 0.01, and ^&&&^*P* < 0.001 compared with the H/R + MTH + si-NC group

SLC25A10 plays a crucial role in many pathophysiological processes, especially in oxidative stress, mitochondria dysfunction, and apoptosis [18]. We next sought to determine whether MTH protects against MI/RI via a SLC25A10-dependent mechanism. First, the expression of SLC25A10 in cardiomyocyte was inhibited by transfection of siRNA targeting SLC25A10 (Fig. 4A). Next, as shown in Fig. 4B–D, treatment of H/R-induced AC16 cells with MTH has significantly restored the increased activity of LDH activity (*p* < 0.001), CK-MB activity (*p* < 0.001), and excessive cTnl (*p* < 0.001), which was partially abolished by the down-regulation of SLC25A10 (*p* < 0.05). Consistently, MTH treatment improved the cell viability inhibited by H/R (*p* < 0.001), while SLC25A10 knockdown suppressed the cell viability as demonstrated by MTT assay (*p* < 0.001; Fig. 4E). These results indicated that MTH treatment could recover the cell injury in H/R cardiomyocytes by targeting SLC25A10.

### MTH Treatment Improved Mitochondrial Dysfunction in H/R Cardiomyocyte by Regulating SLC25A10

**Fig. 5:**
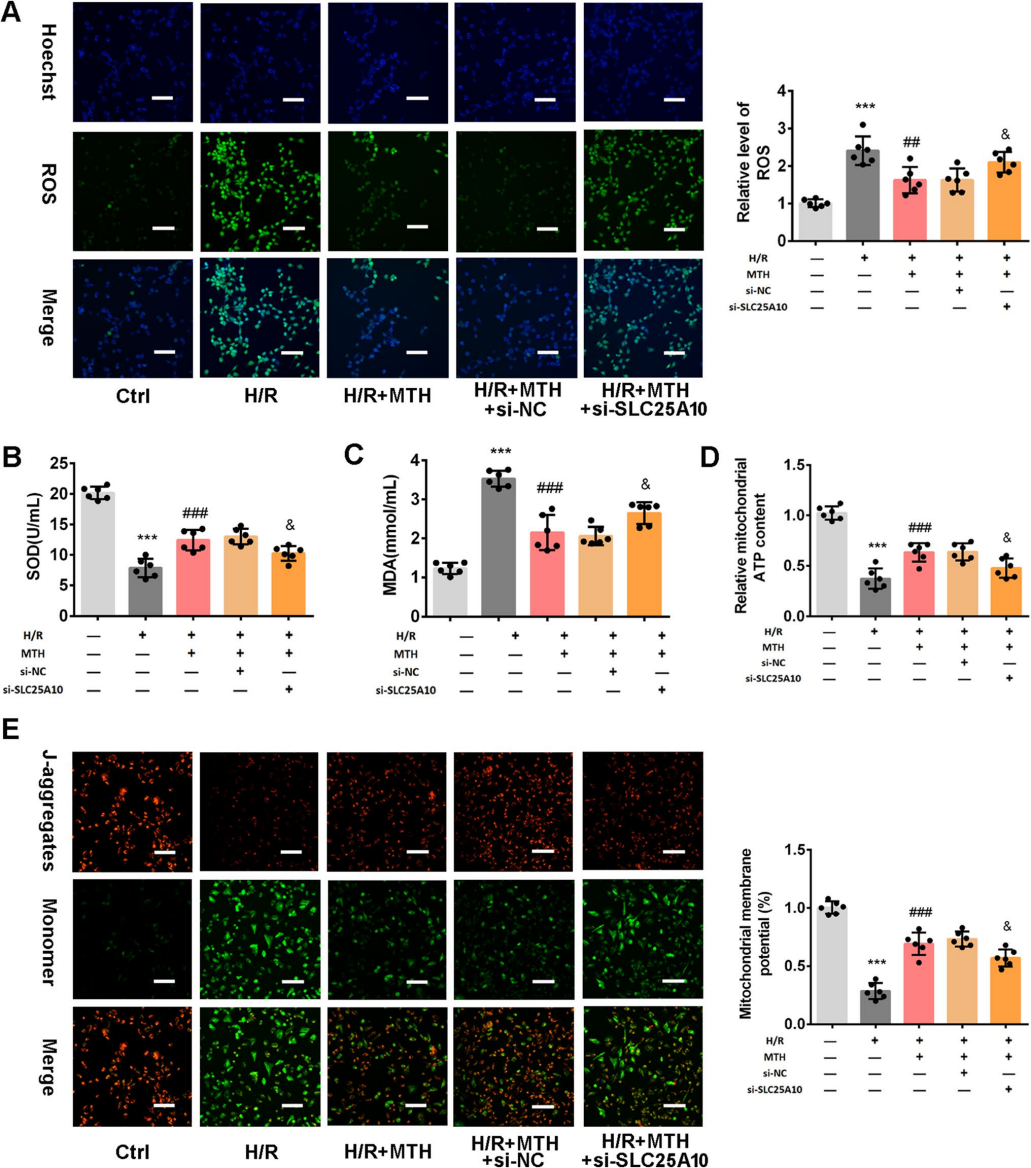
Effects of SLC25A10 on the improvement of mitochondrial function by HM treatment. **A**–**D** Relative level of ROS, SOD activity, MDA content, and ATP content were examined by commercial assay kits. **E** The MMP was examined by JC-1 staining. The red-fluorescent aggregates (Jaggregates) represent high membrane potential; green fluorescence (JC-1 monomers) represents dissipated mitochondrial membrane potential. Scale bar: 100 μm. Data shown are means ± SD, *n* = 6. ***P < 0.001 compared with the Ctrl group; ^##^*P* < 0.01 and ^###^*P* < 0.001 compared with H/R group; ^&^*P* < 0.05 compared with the H/R + MTH + si-NC group

**Fig. 6:**
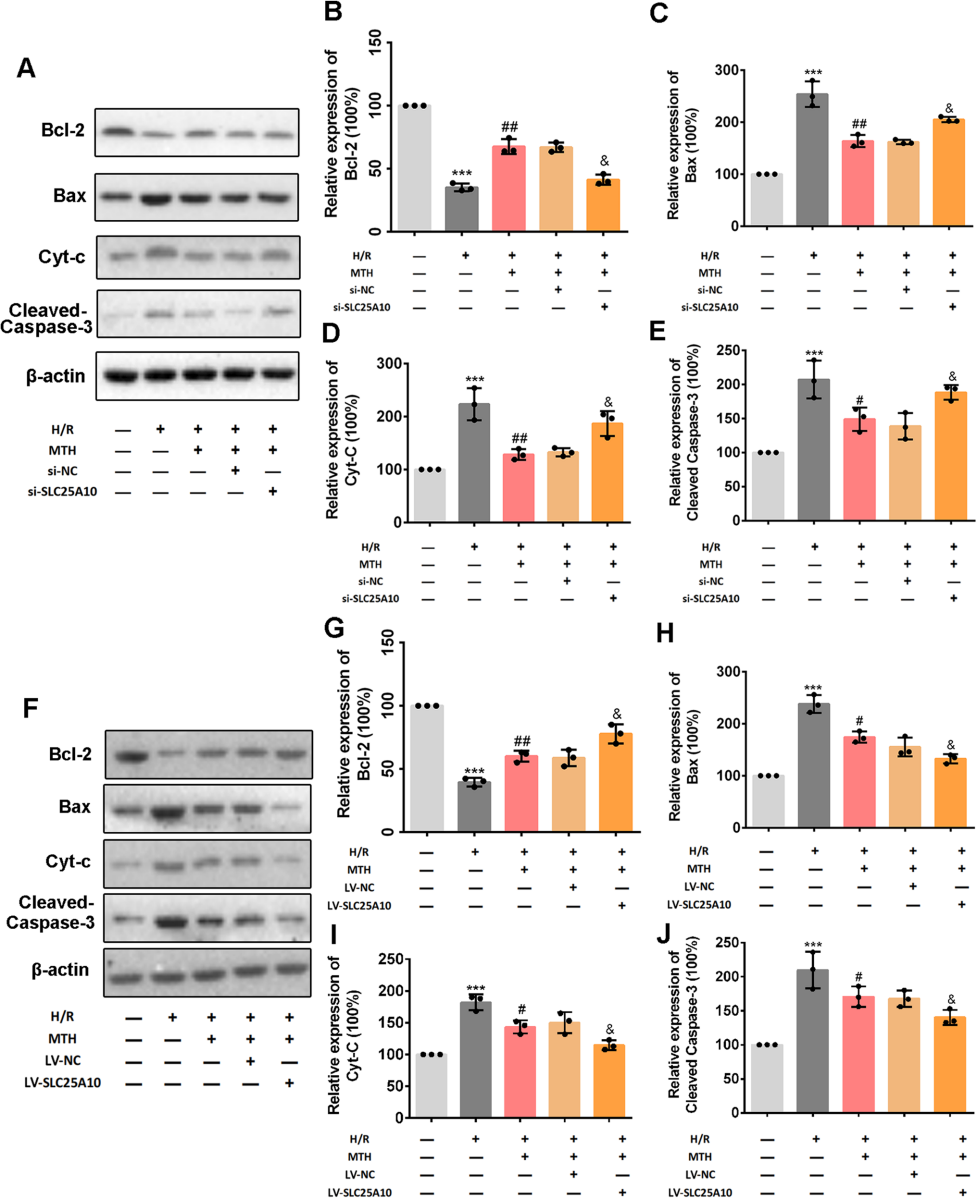
Effects of SLC25A10 on the inhibitory effect of MTH treatment on mitochondria apoptosis pathway in H/R cardiomyocyte. **A**–**J** The expressions of Bcl-2, Bax, Cleaved Caspase-3, and Cyt-C (in cytoplasm) were determined and analyzed by western blot. Data shown are means ± SD, *n* = 3. ^***^P < 0.001 compared with the Ctrl group; ^#^*P* < 0.05 and ^##^*P* < 0.01 compared with the H/R group; ^&^*P* < 0.05 compared with the H/R + MTH + si-NC group or the H/R + MTH + NC group. Schematic diagram of MTH treatment to suppress mitochondrial apoptosis pathway by upregulating SLC25A10 expression in MI/RI

We found that SLC25A10 knockdown partially abolished the inhibitory effect of MTH treatment on oxidative stress, including the relative level of ROS, SOD activity, and MDA content (*p* < 0.05; Fig. 5A–C). Consistently, the results of Fig. 6D–E revealed that H/R significantly reduced intracellular ATP levels and MMP (*p* < 0.001), which were elevated by MTH treatment (*p* < 0.001). However, in SLC25A10 siRNA-treated cells, MTH treatment failed to maintain the improvements in ATP levels and MMP (*p* < 0.05). These results indicated that SLC25A10 might be involved in the protective effect of MTH treatment on mitochondrial function in H/R cells.

### MTH Treatment Suppressed H/R-Induced Mitochondrial Apoptosis in a SLC25A10-Dependent Pathway Mechanism

In the above experimental results, we have confirmed that SLC25A10 activation by MTH treatment significantly improved myocardial cell injury and mitochondrial dysfunction. Here, we further investigated the activation of the mitochondria apoptosis pathway in H/R cardiomyocytes with MTH treatment, SLC25A10 siRNA transfection, or SLC25A10 overexpression vector (Fig. 6). It revealed that MTH treatment effectively reversed the abnormal expressions of Bcl-2 (*p* < 0.01), Bax (*p* < 0.05), Cyt-C (in cytoplasm; *p* < 0.01) and cleaved-caspase-3 (*p* < 0.05), while the inhibitory effects of MTH treatment on mitochondria apoptosis pathway was partially attenuated by SLC25A10 knockdown (*p* < 0.05). However, overexpression of SLC25A10 significantly enhanced the inhibitory effect of MTH on H/R-induced mitochondrial apoptosis pathway (Fig. 6). In addition, SLC25A10 knockdown or expression did not significantly affect the mitochondrial status in cardiomyocytes (Fig. S2). These data suggested that MTH treatment inhibited the mitochondrial apoptotic pathway via targeting SLC25A10.

## Discussion

To date, MTH treatment has already been shown to improve neurological outcome after cardiac arrest and MTH applied during ischemia attenuates cardiomyocyte cell death, but the outcomes of MTH still remain uncertain, leaving its clinical utility inconclusive [19, 20]. Thus, the present study confirmed that MTH treatment significantly improved cardiac function and reduced infarct size, which might be attributable to the inhibition of mitochondrial apoptosis pathway. Further, using nano-UHPLC-MS/MS-based quantitative proteomics, SLC25A10 was identified as an important mediator in the protective beneficial effect of MTH treatment on I/R by modulating mitochondrial function and mitochondrial apoptosis pathway.

In managing AMI, timely reperfusion is essential to rescue the viable myocardium, which is challenged by MI/RI. MI/RI was reported to account for up to 50% of the final infarct size [3]. In fact, continuous efforts have been made to reduce reperfusion injury and improving long-term outcomes [21, 22]. Previous results of animal models suggested that therapeutic hypothermia might reduce MI/RI after urgent revascularization for AMI [23]. Although the therapeutic effects of hypothermia have been documented throughout history, and it was even used clinically as a neuroprotectant in the 1960s [24], the feasibility of TH implementation in the clinical cardiology setting remains uncertain [13, 14, 25]. Herein, by using in vivo and in vitro model, we comprehensively clarified the protective effect of MTH treatment on cardiac function and myocardial infarction during MI/RI.

By nano-UHPLC-MS/MS-based quantitative proteomics, mitochondrial carrier SLC25A10 expressions were dramatically inhibited by MI/RI, which was reversed by MTH treatment in vivo and in vitro. It has been shown that abnormal expression of SLC25A10 would profoundly affect the proliferation of non-small cell lung cancer cells [26]. The function of SLC25A10 is to transport malate and succinate out of the mitochondria in exchange of phosphate, sulfate, and thiosulfate, indicating that altered expression of SLC25A10 might contribute to metabolic reprogramming of cells. Moreover, SLC25A10 was also reported to participate in the maintenance of redox homeostasis. In particular, the reduced expression of SLC25A10 makes cells more vulnerable to oxidative stress and glutamine deprivation [27]. In our study, MTH treatment reduced MI/RI-induced myocardial cell injury by enhancing the expression of SLC25A10. Therefore, we speculated that SLC25A10 might be the key target of MTH treatment for MI/RI.

One of the largest influences of MI/RI on myocardial cell apoptosis is due to mitochondrial failure. Reperfusion to the ischemic myocardium results in a transient burst in ROS production by mitochondria due to the abundant oxygen and up-regulated oxidative phosphorylation [28, 29]. Excessive ROS generation resulted in the oxidation of lipids of plasma and mitochondrial membranes, DNA, and proteins, which induces cardiomyocyte death mainly through the activation of mitochondrial apoptosis pathway [30]. The mitochondrial apoptosis pathway is tightly regulated by a balance of pro-apoptotic and anti-apoptotic proteins, including Bcl-2, Bax, Cyt-C, and caspase-3 [31]. Here, for the first time, we found MTH could reverse mitochondrial dysfunction in H/R cardiomyocytes by reducing ROS release, reversing the ATP level and MMP in a SLC25A10-dependent mechanism. Moreover, the inhibition of MTH treatment on mitochondrial apoptosis pathway in the H/R cardiomyocyte was also dependent on the upregulation of SLC25A10. In addition, we have also tested other pathways responsible for damage caused by MI/RI, including ferroptosis and autophagy and found that MTH treatment or SLC25A10 knockdown did not significantly affect the expressions of ferroptosis and autophagy markers, such as p62 and GPx4 (supplemental Figure S1).

The present study does have some limitations. First, multiple methods have been developed for induction of hibernation-like state. For hibernation-like therapy to be relevant in the clinical treatment, different methods should be compared in terms of high efficacy, minimized adverse effects, and realistic economic feasibility. Moreover, it is not clear whether MTH treatment still could protect against MI/RI in SLC25A10 knockout animals or in MI/RI patients. In addition, lytic cocktail may cause side effects such as dizziness, bloating, delayed reactions, and reduced memory. And there are some potential interactions between MTH and other medications in MI/RI treatment. For example, promethazine may enhance the ulcerogenic effect of potassium chloride [32] and chlorpromazine may enhance the QTc-prolonging effect of Amiodarone [33], which may limit the clinical applications of MTH treatment. Lastly, we only investigated the effects of cooling and not the effects of rewarming on the animals and cardiomyocytes, which may have deleterious side effects. Therefore, future studies need to establish clinical guidelines for the application of MTH treatment.

In summary, we clarified the beneficial effect of MTH treatment on MI/RI, and for the first time, SLC25A10 was identified as the potential target of MTH treatment in reversing the mitochondrial apoptosis. Our findings not only provide new insights in understanding the mechanism of MI/RI but also suggested that regulation of SLC25A10 could be a potential intervention to enhance the clinical utility of MTH treatment.

## Supplementary Information

Below is the link to the electronic supplementary material.

Supplementary file1 (PDF 83 KB)

Supplementary file2 (PDF 76 KB)

## Data Availability

The data that support the findings of this study are available from the corresponding author upon reasonable request.

## Abbreviations

AMI: Acute myocardial infarction
ATP: Adenosine triphosphate
CK: Creatine kinase
IHD: Ischemic heart disease
cTnl: Cardiac troponin 1
Cyt-C: Cytochrome C
H/R: Hypoxia and reoxygenation
I/R: Ischemia/reperfusion
JC-1: 5,5′,6,6′-Tetrachloro-1,1′,3,3′-tetraethylbenzimid azolcarbocyanine iodide
LDH: Lactic dehydrogenase
LVIDd: Left ventricular internal dimension at end-diastole
LVIDs: Left ventricular internal dimension at systole
MDA: Malondialdehyde
MI/RI: Myocardial ischemia/reperfusion injury
MMP: Mitochondrial membrane potential
MS/MS: Tandem mass spectrometry
MTH: Mild therapeutic hypothermia
PLS-DA: Partial least squares discriminant analysis
ROS: Reactive oxygen species
SD: Sprague-Dawley
SOD: Superoxide dismutase
TTC: Triphenyl tetrazolium chloride
UHPLC: Ultra-high performance liquid chromatography

## Declarations

### Animal Studies

All institutional and national guidelines for the care and use of laboratory animals were followed and approved by the appropriate institutional committees. All procedures were approved by the ethics committee of Department of Experimental Animal Science, Fudan University (2022JS Huashan Hospital-070).
