# Supplementary material for: Mild Therapeutic Hypothermia Alleviated Myocardial Ischemia/Reperfusion Injury via Targeting SLC25A10 to Suppress Mitochondrial Apoptosis
Source: J Cardiovasc Transl Res. 2024 Apr 3;17(4):946–58. doi: 10.1007/s12265-024-10503-z (PMC11371862; doi:10.1007/s12265-024-10503-z)
Supplement: Supplementary file 1 — Supplementary file1 (PDF 83 KB) [file 12265_2024_10503_MOESM1_ESM.pdf]

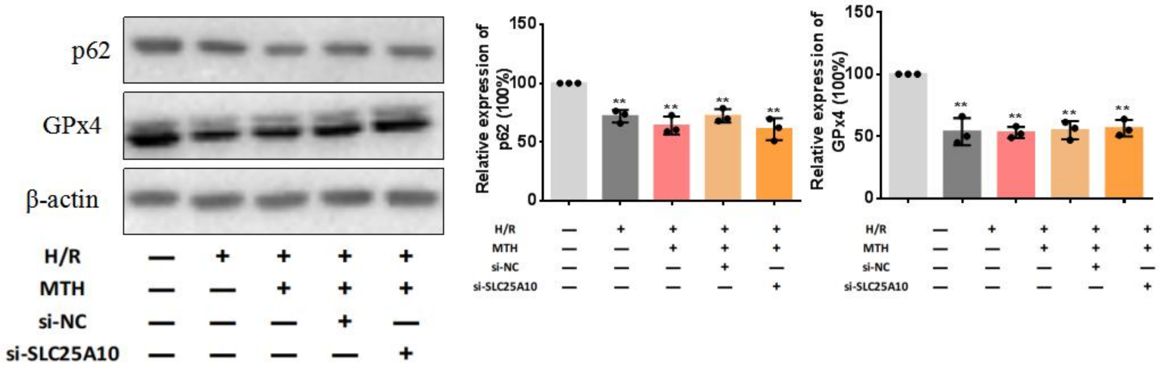

Fig. S1 Effects of MTH treatment or SLC25A10 knockdown on ferroptosis and autophagy. The markers, such as p62 and GPx4, were determined and analyzed by western blot. Data shown are means ± SD, n=3. **P < 0.01 compared with Ctrl group.

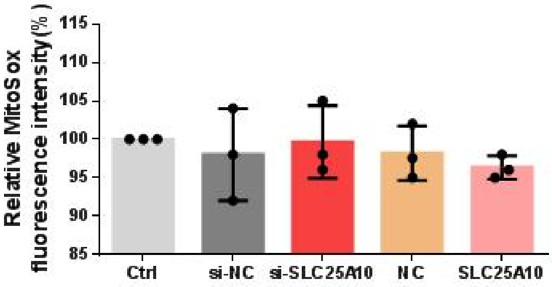

Fig. S2 Effects of SLC25A10 expressions on mitochondrial status in cardiomyocytes were determined by MitoSox staining.
