# Supplementary material for: Mild Therapeutic Hypothermia Alleviated Myocardial Ischemia/Reperfusion Injury via Targeting SLC25A10 to Suppress Mitochondrial Apoptosis
Source: J Cardiovasc Transl Res. 2024 Apr 3;17(4):946–58. doi: 10.1007/s12265-024-10503-z (PMC11371862; doi:10.1007/s12265-024-10503-z)
Supplement: Supplementary file 2 — Supplementary file2 (PDF 76 KB) [file 12265_2024_10503_MOESM2_ESM.pdf]

Table S1. Differentiated expression proteins in sham group compared with I/R group

PG.ProteinGroups	PG.Genes	PG.ProteinDescriptions
P49301;Q5BKA0	Clec10a	C-type lectin domain family 10 member A
D3Z881;D3ZGN0	Tbc1d4	TBC1 domain family, member 4;TBC1 domain family, member 4
Q6KC51	Ablim2	Actin-binding LIM protein 2
F1LMV6	Dsp	Desmoplakin
A0A0G2JVW5	Huwe1	E3 ubiquitin-protein ligase HUWE1
A0A0G2JZI2	Eprs	Glutamyl-prolyl-tRNA synthetase
O89035	Slc25a10	Mitochondrial dicarboxylate carrier
D3ZF12	Spcs3	Signal peptidase complex subunit 3
D3ZWS0;G3V6R4	Scrib;Lrrc1	Scribbled planar cell polarity protein;Leucine-rich repeat-containing 1
A0A0G2JY08;D3ZFD0	Myo18a	Myosin XVIIIa
D3ZPF0	Fhl3	Four and a half LIM domains 3
B2RYI2	Srp68	Signal recognition particle subunit SRP68
A0A0G2JT45;Q5XIJ7	Cab39l	Calcium-binding protein 39-like
D3ZAS9	Ddrgk1	DDRGK domain-containing 1
Q6MGB8	RT1-A2	RT1 class I, A2
A0A0G2JXP4;D3ZY86	Tango2	Transport and golgi organization 2 homolog;Similar to Ser/Thr-rich protein T10 in DGCR region (Predicted), isoform CRA_a
D3ZHY9	Rasal1	RAS protein activator like 1 (GAP1 like) (Predicted)
A0A140TAE6;Q9Z311	Mecr	Enoyl-[acyl-carrier-protein] reductase, mitochondrial;Enoyl-[acyl-carrier-protein] reductase, mitochondrial
Q64350	Eif2b5	Translation initiation factor eIF-2B subunit epsilon
O35092	Timm17a	Mitochondrial import inner membrane translocase subunit Tim17-A
Q7TP77	Mrpl49	Aa2-277
P52944	Pdlim1	PDZ and LIM domain protein 1
Q6AYI1	Ddx5	DEAD (Asp-Glu-Ala-Asp) box polypeptide 5
D4A7X5	Ppm1k	Protein phosphatase 1K (PP2C domain containing) (Predicted)
F1LVF4	Cox10	Protoheme IX farnesyltransferase, mitochondrial
A0A0H2UI06;D3ZN43	Ndufaf6	NADH dehydrogenase (ubiquinone) complex I, assembly factor 6;NADH dehydrogenase (ubiquinone) complex I, assembly factor 6
E9PU29	Rnf31	Ring finger protein 31
F1LML7	Hip1r	Huntingtin-interacting protein 1-related
A0A0G2K3P9;Q6	Syngr1	Synaptogyrin-1 (Fragment);Synaptogyrin-1

2876		
D4A720	Srsf7	RCG61762, isoform CRA_d
D3ZER6	Tnpo2	Transportin 2
B0BNJ9	Tmem14c	RCG44002, isoform CRA_a
Q5XIN4	Mtmr9	Myotubularin-related protein 9
P24268;Q6P6T6	Ctsd	Cathepsin D
Q5BJX0	Ntmt1	N-terminal Xaa-Pro-Lys N-methyltransferase 1
P47860	Pfkp	ATP-dependent 6-phosphofructokinase, platelet type
D3ZS75	Ndufc1	NADH:ubiquinone oxidoreductase subunit C1
A0A0G2K876;F8WFW5;Q01062	Pde2a	Phosphodiesterase;Phosphodiesterase;cGMP-dependent 3',5'-cyclic phosphodiesterase
D4ACR1		Uncharacterized protein

Table S2. Differentiated expression proteins in MTH+I/R group compared with I/R group

PG.ProteinGroups	PG.Genes	PG.ProteinDescriptions
D3Z8I7	Gstt3	Glutathione S-transferase, theta 3
D3ZZL5	Gpihbp1	Glycosylphosphatidylinositol-anchored high density lipoprotein-binding protein 1
A0A0G2K7C6;P35815;Q642F2;Q99ND8	Ppm1b	Protein phosphatase 1B;Protein phosphatase 1B;Ppm1b protein;Ppm1b protein
Q6AY80	Nqo2	Ribosyldihydronicotinamide dehydrogenase [quinone]
G3V940	Coro1b	Coronin
Q5FVQ9	Tbce	Tubulin-specific chaperone E
B2RYW8	Minos1	MICOS complex subunit Mic10
F1LML7	Hip1r	Huntingtin-interacting protein 1-related
Q6Q7Y5	Gna13	Guanine nucleotide-binding protein subunit alpha-13
B1H248;P0C0A2	Vps36	Vacuolar protein-sorting-associated protein 36
P52944	Pdlim1	PDZ and LIM domain protein 1
A0A0G2K0B4;A0A0G2K8P3	Nedd4	E3 ubiquitin-protein ligase
O35092	Timm17a	Mitochondrial import inner membrane translocase subunit Tim17-A
Q9Z2Q7	Stx8	Syntaxin-8
Q9JI04	Col5a3	Alpha 4 type V collagen
D3ZRM0	Dusp27	Dual-specificity phosphatase 27, atypical
A0A0G2JU49;B0BN02	Mtx1	Metaxin 1
O89035	Slc25a10	Mitochondrial dicarboxylate carrier
Q63610	Tpm3	Tropomyosin alpha-3 chain
P30835	Pfkl	ATP-dependent 6-phosphofructokinase, liver type
A0A0A0MXU2;A0A0G2K9F9;O3	Coro7	Coronin;Coronin;Coronin-7
